# DNA methylation accelerated age as captured by epigenetic clocks influences breast cancer risk

**DOI:** 10.3389/fonc.2023.1150731

**Published:** 2023-03-15

**Authors:** Celina I. Valencia, Devin Saunders, Jennifer Daw, Adria Vasquez

**Affiliations:** ^1^ Department of Family and Community Medicine, College of Medicine—Tucson, University of Arizona, Tucson, AZ, United States; ^2^ Department of Nutritional Sciences, College of Agriculture and Life Sciences, University of Arizona, Tucson, AZ, United States; ^3^ Cancer Biology Program, College of Medicine, University of Arizona, Tucson, AZ, United States; ^4^ Department of Health Sciences, Bloomberg School of Public Health, Johns Hopkins University, Baltimore, MD, United States

**Keywords:** epigenetic clocks, accelerated age, DNAm, breast cancer, underrepresented populations

## Abstract

**Introduction:**

Breast cancer continues to be the leading form of cancer among women in the United States. Additionally, disparities across the breast cancer continuum continue to increase for women of historically marginalized populations. The mechanism driving these trends are unclear, however, accelerated biological age may provide key insights into better understanding these disease patterns. Accelerated age measured by DNA methylation using epigenetic clocks is to date the most robust method for estimating accelerated age. Here we synthesize the existing evidence on epigenetic clocks measurement of DNA methylation based accelerated age and breast cancer outcomes.

**Methods:**

Our database searches were conducted from January 2022 to April 2022 and yielded a total of 2,908 articles for consideration. We implemented methods derived from guidance of the PROSPERO Scoping Review Protocol to assess articles in the PubMed database on epigenetic clocks and breast cancer risk.

**Results:**

Five articles were deemed appropriate for inclusion in this review. Ten epigenetic clocks were used across the five articles demonstrating statistically significant results for breast cancer risk. DNA methylation accelerated age varied by sample type. The studies did not consider social factors or epidemiological risk factors. The studies lacked representation of ancestrally diverse populations.

**Discussion:**

DNA methylation based accelerated age as captured by epigenetic clocks has a statistically significant associative relationship with breast cancer risk, however, important social factors that contribute to patterns of methylation were not comprehensively considered in the available literature. More research is needed on DNA methylation based accelerated age across the lifespan including during menopausal transition and in diverse populations. This review demonstrates that DNA methylation accelerated age may provide key insights for tackling increasing rates of U.S. breast cancer incidence and overall disease disparities experienced by women from minoritized backgrounds.

## Introduction

Despite advances in breast cancer screening technologies and extensive research on the disease, breast cancer persists as having the heaviest cancer burden in women in the United States (U.S.) with marked disease disparities occurring in minoritized populations. Since 2004, there has been a 0.4% rate increase of breast cancer incidence with estimates suggesting 1 in 8 women will be affected by this diagnosis in their lifetime (1). Over the last decade the rate of early onset breast cancer, diagnosis before the age of 50, has increased significantly (2, 3). Early onset breast cancer is often a more aggressive disease type, is diagnosed at a later stage, and the prognosis is often poor (2). Patients that survive early onset breast cancer are faced with different survivorship issues impacting their quality of life (4). Increasing breast cancer rates, particularly early onset disease, is a pressing public health issue that requires new clinical and translational approaches for curbing these disease trends. A closer consideration of accelerated biological age may provide promising avenues for disease prevention, particularly for early onset breast cancer.

Age remains one of the strongest predictors of breast cancer (5, 6) making it unclear why increasing rates of early onset breast cancer is occurring in the U.S. One potential pathway for understanding the growing trend of early onset breast cancer is identifying the role of accelerated biological age in breast cancer risk. Biological age is marked by progressive declines in the body’s systems also referred to as the hallmarks of aging (7, 8) and these declines increase vulnerability to disease and death. Of the nine hallmarks of aging (7, 8), seven have been implicated in the development of breast cancer, these markers are: genomic instability (9), telomere attrition (9), epigenetic alterations (10), deregulated nutrient-sensing (11, 12), mitochondrial dysfunction (12), cellular senescence (11), and altered intercellular communication (11). The findings of these shared molecular hallmarks in predicting breast cancer risk have been contradictory (9, 11).

**Figure 1:**
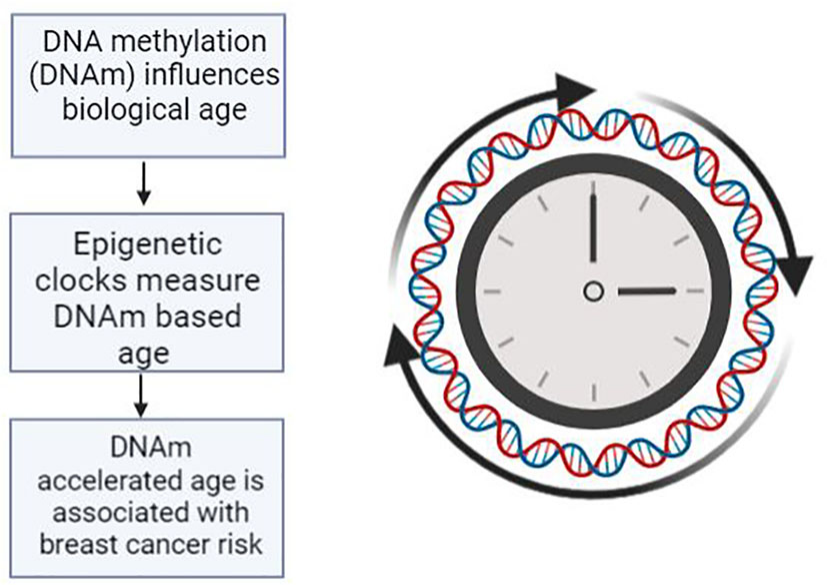
Breast Cancer riskis influenced by biological age measured by epigenetic clocks.

To date, the epigenetic alteration of DNA methylation is the most robust predictor of biological age (13, 14). DNA methylation is an epigenetic marker that occurs on cytosine nucleotides most often in the context of CpG (cytosine-phosphate-guanine) islands and often correlates with age (15). DNA methylation (DNAm) is an established hallmark of oncogenesis and pathophysiology of cancer progression (14). The consideration of the difference between biological age and chronological age as captured by DNAm, and the increased risk for cancer development *via* DNAm provides a key site of inquiry for disease prevention. This review focuses on elucidating the available evidence on DNAm in breast cancer risk as a potential marker for surveillance and intervention as DNAm has been found to be reversible and modifiable *via* lifestyle and psychological intervention (16, 17) (**Figure 1**).

Epigenetic clocks were developed using machine learning to create multivariate weighted sums of DNA methylation at CpG sites across the genome to assess biological age (14). Three distinct biological processes are captured by this measurement tool: DNAm-based age estimator (13), the physiological process of aging (14), and the body’s sensitivity to social adversity (18–20). Epigenetic clocks categorized as second-generation have integrated clinical biomarkers that are surrogates of stress into their algorithm to assess healthspan whereas first-generation clocks predicted lifespan (13). They have been found to be a predictive tool for various health outcomes including cancer, menopausal timing, and mortality (21). The prediction capacity for menopausal timing, as well as cancer and mortality, is particularly salient for breast cancer as menopause is used as a delineating factor within the disease, pre- and post-menopausal, and these categories are affiliated with different epidemiological risk factors. As a predictor for breast cancer, DNAm has had substantial translational applications to address issues of poor diagnostics and identifying clinical biomarkers of disease (22), however, the interaction of race and social environments in epigenetic clocks for disease risk have not been comprehensively considered (23).

Here our review demonstrates DNAm based accelerated age as captured by epigenetic clocks has an associative relationship with breast cancer risks, however, important social factors that contribute to patterns of methylation were not considered in these studies. The included studies showed that DNAm accelerated age during critical life stages such as the menopausal transition may play a role in the development of breast cancer. None of the included studies considered methylation patterns over time limiting our ability to assess the magnitude of fluctuating DNAm accelerated aging over time. The studies did not consider the role of social adversity, an established factor in patterns of methylation (5), on DNAm accelerated age leaving a gap in our understanding of the interplay of social adversity driven DNAm accelerated age and breast cancer risk. Additionally, the studies lacked inclusion of ancestrally diverse individuals making it difficult to discern the race and social environment interaction on methylation patterns that may shape breast cancer disparities, particularly in early onset disease. The findings of the review demonstrate that epigenetic clocks provide a useful tool for tackling the trend of increasing breast cancer incidence in the U.S. and may provide key insights to better understand disease disparities. However, more research is needed on social factors of methylation and epigenetic clock measurement.

## Methods

We developed our methods and approach using guidance provided by the PROSPERO Scoping Review Protocol to ensure a rigorous literature review. Database search terms specifically for PubMed were developed in consultation with a research librarian. The search term language is as follows: (“Breast Neoplasms”[Majr] OR breast cancer [tiab] OR breast cancers [tiab]) AND (“Epigenesis, Genetic”[Mesh] or “DNA Methylation” [mesh] OR “Epigenomics”[Mesh] OR epigenesis [ti] OR epigenetic [ti] OR epigenetics [ti] OR dna methylation [tiab] OR BeadChip* [tiab]) AND (“risk factors” [mesh] or “risk” [mesh] OR “risk assessment” [mesh] OR “genetic predisposition to disease” [mesh] OR risk [tiab] OR risks [tiab] OR predictor* [tiab] OR association [tiab] OR correlat* [tiab] OR clock [tiab] OR clocks [tiab]). A total of seven searches of PubMed using search terms were completed by study team members from January 2022 to April 2022. The searches yielded a total of 2,908 articles. Seventy-five articles were identified as suitable for additional review and discussion for inclusion in this review.

The eligibility for inclusion was: 1) the study must use an epigenetic clock to assess epigenetic accelerated age in their sample, 2) primary outcome is breast cancer risk, 3) U.S. based study sample 4) published in a peer-reviewed PubMed indexed journal, 5) must have been published by April 30, 2022. Three individuals (AV, DS, and CIV) independently conducted reviews of all titles and abstracts yielded in the searches to assess the inclusion and exclusion criteria of each article. In the first round of reviews duplicate articles were identified and removed. During the independent reviews, the three individuals made decisions on the reviewed articles eligibility for inclusion. Articles that had conflicting decisions for inclusion or exclusion made during the independent reviews were discussed by the reviewers (AV, DS, CIV). Final decisions were made on the article with conflicting decisions based on consensus. In instances where consensus could not be reached, CIV made the final decision for inclusion. Through this process a total of five articles were identified as suitable for inclusion in the review.

Following the team decision phase, articles identified as being appropriate for inclusion were then examined for data extraction. Data extraction was conducted using a uniform extraction tool that identified the research design, study sample, specimen type, analysis conducted, and study results. Data extraction and coding was conducted by DS, AV, and CIV. Next, we completed a data summary phase conducted by DS and CIV. As the studies included in the review were subjected to the peer review process of a PubMed indexed journal, this process was assumed to provide a robust critical appraisal of the scientific product within the studies in this review. Lastly, an additional level of scrutiny was applied to articles *via* discussions between DS, JD, and CIV to identify recurring themes and methods across the studies. The identified themes of the included studies are outlined in the results section.

## Results

To assess the evidence on the utility of epigenetic clocks in predicting breast cancer risk, we used the PROSPERO Scoping Review Protocol for guidance on conducting a high-quality review. We first conducted a comprehensive search on the PubMed database to capture relevant articles. We then independently reviewed and applied the outlined inclusion and exclusion criteria to the articles yielded in the database search. We extracted the data for the studies deemed appropriate for inclusion for this review. Lastly, we synthesized the findings of the included papers to identify important evidence and themes. A total of five articles met the inclusion criteria (**Table 1**) (24–28).

### Studies lacked ancestrally diverse samples and social risk factors

The data sources used by these studies included Komen Breast Tissue Bank (26, 28), the prospective cohort Sister Study (25, 27), National Cancer Institute Genomic Data Commons (24), and samples from clinical settings (24). The study of the Sister Study (25) had the largest sample sizes of N=2,764. The rest of the studies analyzed data from observational and clinical sources with sample sizes. The smallest sample size was found in the study conducted by Hofstatter et al. (28), with a sample size of N=88. Blood and breast tissue samples were the most frequently used biospecimen (**Table 1**). Non-Hispanic White (NHW) is the population represented in the highest numbers across all five studies, with the most diverse sample found in Rozenblit et al. (26) at 75% NHW reporting an inclusion of 178 African American women (n=178). The design of the majority of studies included was case control with only the Sister Study cohort study providing an opportunity to consider the role of epidemiological risk factors and patterns of methylation over time. Time varying analysis would provide important insights on crucial surveillance periods to reduce breast cancer risk. Breast cancer risk factors that are also epigenetic age accelerators like socioeconomic position (5, 20) were not considered in the included studies.

**Table 1:** Studies Included in Review.

Source	Study Design	Data Source	Tissue Sample	Epigenetic Clock	Outcomes
Rozenblit M, Hofstatter E, Liu Z, et al. Evidence of accelerated epigenetic aging of breast tissues in patients with breast cancer is driven by CpGs associated with polycomb-related genes. *Clin Epigenetics*. 2022;14(1):30. Published 2022 Feb 24. doi:10.1186/s13148-022-01249-z	Case-control (N=270)	4 cohort studies	Peripheral blood Breast tissue	Horvath Hannum Levine Horvath 2 Lin Yang	All epigenetic clocks strongly associated with breast cancer status
		75% Non-Hispanic White women			Levine and Yang clocks were associated with breast cancer and showed significant age acceleration
Kresovich JK, Xu Z, O'Brien KM, Weinberg CR, Sandler DP, Taylor JA. Methylation-Based Biological Age and Breast Cancer Risk. *J Natl Cancer Inst*. 2019;111(10):1051-1058. doi:10.1093/jnci/djz020	Case-cohort (n=1289)	Sister Study	Blood DNAm	Hannum	All clocks were strongly correlated with chronological age (p <.001)
		Non-Hispanic White women		Horvath (multi-tissue)	All clocks had statistically significant association with breast cancer risk
		Ages 35-75 years old		Levine PhenoAge	Levine PhenoAge had best fit (p <.001)
Kresovich JK, Xu Z, O'Brien KM, Weinberg CR, Sandler DP, Taylor JA. Epigenetic mortality predictors and incidence of breast cancer. *Aging (Albany NY)*. 2019;11(24):11975-11987. doi:10.18632/aging.102523	Case-cohort (n=1294)	Sister Study	Whole blood sample	GrimAge	Clocks were not associated with breast cancer incidence
		Non-Hispanic White women		Mortality Score	Invasive and ductal carcinoma in situ one positively but weakly associated with GrimAge
		Ages 35-75 years old			
Hofstatter EW, Horvath S, Dalela D, et al. Increased epigenetic age in normal breast tissue from luminal breast cancer patients. *Clin Epigenetics*. 2018;10(1):112. Published 2018 Aug 29. doi:10.1186/s13148-018-0534-8	Case-control (N=88)	Komen Tissue Bank	Breast tissue	DNAmAge	Epigenetic age acceleration in non-cancerous breast tissue in patients with luminal breast cancer was significantly higher than in unaffected women
		91.4% Non-Hispanic White women			
Ren JT, Wang MX, Su Y, Tang LY, Ren ZF. Decelerated DNA methylation age predicts poor prognosis of breast cancer. *BMC Cancer*. 2018;18(1):989. Published 2018 Oct 17. doi:10.1186/s12885-018-4884-6	Secondary data analysis (N=1085)	Genomics Common Data 83.5% Non-Hispanic White women	Breast tissue	Horvath	Younger DNAm age in cancerous tissues of breast predicted poorer prognosis in the sample

### Epigenetic clocks measurement of DNAm accelerated age was associated with breast cancer

The studies included in this review indicate that DNAm accelerated age as measured by epigenetic clocks had an associative relationship with the development of breast cancer. This indicates that DNAm accelerated age may serve as an important and understudied disease risk factor. A total of 10 different epigenetic clocks were used (**Table 1**). The Horvath clock was most frequently used appearing in three of the five studies (24–26). The included studies found that DNAm age from the following epigenetic clocks had a statistically significant association with breast cancer: Hannum (25), Horvath (25), Levine (25, 26), GrimAge (25, 27), DNAmAge (28), and Yang (26).

### DNAm accelerated age correlates with chronological age and varied by biosample type

The studies determined there was a correlation between DNAm measured by epigenetic clocks and chronological age. Ren et al. (24) applied the Horvath clock to data from the Genomic Data Commons (N=1076) and had a high correlation of DNAm and chronological age (*r*=0.96). Kresovich et al. (25) using the Sister Study (N=2,764) found correlations with Hannum (*r*=0.88), Horvath (*r*=0.87) and Levine (*r*=0.83) DNAm age and chronological age. Rozenblit et al. (26) tested for correlations of DNAm age and chronological age in both cases and controls. For breast tissue (n=84), five of the six clocks used showed significant correlations ranging from the Levine clock *r*=0.35 to the Lin clock *r*=0.68. In peripheral blood (n=170) all six epigenetic clocks DNAm correlated with chronological age (17).

Studies conducted by Ren et al. (24), Rozenblit et al. (26), Hofstatter et al. (28), showed there were differences in DNAm accelerated age based on the type of sample where the epigenetic clock was applied. Ren et al. (24) found that there was a deaccelerated, or younger, DNAm age in the malignant breast tissue versus non-malignant breast tissue and the younger DNAm age predicted a poorer prognosis. The Pearson coefficient for between DNAm and chronological age were *r*= 0.85 (*p <*0.01) for normal breast tissue and *r*=0.30 (*p <*0.01) for malignant breast tissue (24). Hofstatter et al. (28) (N=88) demonstrated the epigenetic age in the non-tumor tissue of women with luminal breast cancer was substantially higher than the breast tissue of women without breast cancer. In the age-matched comparison normal breast tissue the normal tissue of the breast cancer patient was approximately five years older, or more, which the study attributed to treatment effects (28). The study conducted by Rozenblit et al. (26) found that peripheral blood (n=170) has a higher age correlation than was observed in breast tissue (n=84). Additionally, Rozenblit et al.’s (26) findings showed that accelerated DNAm in the non-malignant breast tissues of women with breast cancer have methylation signatures that more closely resemble tumors than the breast tissue of the women without cancer.

Kresovich et al. (25) identified a period of age acceleration predating menopausal transition as an independent risk factor for breast cancer in the Sister Study sample. Ren et al. (24) findings also indicated that breast cancer patients that were premenopausal had DNAm age that was decelerated. The other studies included in this review examined the DNAm age in the period post-breast cancer diagnosis. More research is needed that examines periods of DNAm age acceleration across the lifespan that may signal breast cancer risk.

## Discussion

Our review identifies a gap in the literature on the assessment of social factors, such as adversity and epidemiological risk factors, as a driver of DNAm accelerated age measured by epigenetic clocks. This is a critical gap as there is substantial overlap in factors that result in DNAm age acceleration (5) and breast cancer risk (29). As a predictor for breast cancer, DNAm has substantial translational applications to address current issues of poor diagnostics and identifying clinical biomarkers of disease (22) the addition of social factors in the assessments of DNAm and breast cancer risk can provide meaningful insights for curbing patterns of disease disparities (30).

Epigenetic clocks have been developed to be applied to different biosamples such as tissue, blood, saliva, and cells such as buccal epithelial cells (31). Comparison of malignant and non-malignant tissue within the samples of the included studies showed varying DNAm ages (24, 26). Ren et al.’s (24) and Hofstatter et al. (28) studies demonstrate that there is a difference in DNAm accelerated age in normal breast tissue versus malignant breast tissue. Rozenblit et al. (26) also found that peripheral blood had a different and higher DNAm age than the breast tissue. The variance in DNAm age may indicate that the different biosamples are indicating different mechanisms of biological aging, a larger question currently being investigated in the field of epigenetics (5). These differences in samples being used complicate our ability to compare across different studies. As epigenetics is a nascent science, the field would benefit from the establishment of uniform approaches to standardize evidence and validate findings for comparison allowing for more generalizability.

Menopausal transition and menopause are major milestones in women’s lives with health implications that extend beyond reproductive capacity (32). The importance of time periods surrounding menopause was highlighted in the work of Kresovich et al. (25). Previous evidence has indicated women with late menopause onset were epigenetically younger than women with early menopause onset (33). Timing of menopause has been found to occur earlier in Black women (34) and Latina women (35). The variance of menopausal transition and onset may be the result of social positionality based on socioeconomic factors, exposure to adversity, and environmental factors (34, 35). More evidence is needed to begin to disentangle the associative relationship of these constructs to better understand these complex and overlapping drivers of methylation and breast cancer.

The data gap of ancestrally diverse populations in human genetic research has been previously discussed (19). Validation studies available on epigenetic clocks have demonstrated inconsistent predictive capabilities in ancestrally diverse populations (23). The inconsistency of predictive capacity of epigenetic clocks in ancestrally diverse populations may arise from cumulative biological impact of chronic exposure to socially structured stressors tied to race/ethnicity that would not be captured in NHW the population most often represented in epigenetic clock studies (23). In the U.S., minoritized women experience greater disparities and face more negative breast cancer outcomes, including a higher rate of mortality (36), which are often attributed to various social determinants of health (29, 37–40). A better understanding of the function of DNAm accelerated age in minoritized women may provide key insights into the role of biological age in the patterns of breast cancer disparities across minoritized women in the U.S. As the samples in these studies demonstrate, there is a critical need for more studies in this area that focus on diverse populations.

This review paper comprehensively syntheses the available evidence on epigenetic clocks in breast cancer to understand the role of DNAm accelerated age in breast cancer risk with the goal of identifying translational tools to target disparities experienced by minoritized women. Epigenetic clocks are a promising tool for breast cancer risk surveillance and should be evaluated for integration into clinical practices for disease prevention intervention which could bring us closer to alleviating the overall breast cancer burden experienced by women. As DNAm is reversible and modifiable through psychological (16, 41, 42) and lifestyle interventions (17), more evidence on the role of DNAm in diverse populations may provide new opportunities for intervention targeting breast cancer disparities. Future research on epigenetic accelerated age, as measured by epigenetic clocks, is an important consideration towards better understanding breast cancer risk and disparities. More research is needed to expand our understanding of the role of epigenetic accelerated age in early onset breast cancer, periods of epigenetic acceleration across the lifespan, the role of social factors on DNAm accelerated age in women with breast cancer, and more inclusive and diverse study samples.

## Author contributions

CV, DS, JD contributed to conception and design of study. CV, DS, JD, AV contributed to the database searches and article reviews. CV wrote the first draft of the manuscript. All authors contributed to manuscript revision, read, and approved the submitted version.

## References

[1] DeSantis CE Ma J Gaudet MM Newman LA Miller KD Goding Sauer A. Breast cancer statistics, 2019. CA: Cancer J Clin (2019) 69(6):438–51. doi: 10.3322/caac.21583 31577379

[2] Kudela E Samec M Kubatka P Nachajova M Laucekova Z Liskova A. Breast cancer in young women: status quo and advanced disease management by a predictive, preventive, and personalized approach. Cancers. (2019) 11(11):1791. doi: 10.3390/cancers11111791 31739537 PMC6896106

[3] Fröhlich H Patjoshi S Yeghiazaryan K Kehrer C Kuhn W Golubnitschaja O. Premenopausal breast cancer: potential clinical utility of a multi-omics based machine learning approach for patient stratification. EPMA J (2018) 9(2):175–86. doi: 10.1007/s13167-018-0131-0 PMC5972143 29896316

[4] Cathcart-Rake EJ Ruddy KJ Bleyer A Johnson RH. Breast cancer in adolescent and young adult women under the age of 40 years. JCO Oncol Practice. (2021) 17(6):305–13. doi: 10.1200/OP.20.00793 33449828

[5] Oblak L van der Zaag J Higgins-Chen AT Levine ME Boks MP. A systematic review of biological, social and environmental factors associated with epigenetic clock acceleration. Ageing Res Rev (2021) 69:101348. doi: 10.1016/j.arr.2021.101348 33930583

[6] Freedman RA Keating NL Lin NU Winer EP Vaz-Luis I Lii J. Breast cancer-specific survival by age: Worse outcomes for the oldest patients. Cancer. (2018) 124(10):2184–91. doi: 10.1002/cncr.31308 PMC5935594 29499074

[7] Lemoine M. The evolution of the hallmarks of aging. Front Genet (2021) 12:693071. doi: 10.3389/fgene.2021.693071 34512720 PMC8427668

[8] López-Otín C Blasco MA Partridge L Serrano M Kroemer G. The hallmarks of aging. Cell. (2013) 153(6):1194–217. doi: 10.1016/j.cell.2013.05.039 PMC3836174 23746838

[9] Giaccherini M Gentiluomo M Fornili M Lucenteforte E Baglietto L Campa D. Association between telomere length and mitochondrial copy number and cancer risk in humans: a meta-analysis on more than 300,000 individuals. Crit Rev Oncology/Hematology. (2021) 167:103510. doi: 10.1016/j.critrevonc.2021.103510 34695574

[10] Hanahan D. Hallmarks of cancer: new dimensions. Cancer discovery. (2022) 12(1):31–46. doi: 10.1158/2159-8290.CD-21-1059 35022204

[11] López-Otín C Blasco MA Partridge L Serrano M Kroemer G. Hallmarks of aging: An expanding universe. Cell (2013) 153(6):1194–217. doi: 10.1016/j.cell.2013.05.039 PMC3836174 23746838

[12] Aunan JR Cho WC Søreide K. The biology of aging and cancer: a brief overview of shared and divergent molecular hallmarks. Aging disease. (2017) 8(5):628. doi: 10.14336/AD.2017.0103 28966806 PMC5614326

[13] Palma-Gudiel H Fañanás L Horvath S Zannas AS. Chapter five - psychosocial stress and epigenetic aging. In: Clow A Smyth N, editors. International review of neurobiology, vol. 150. Cambridge, Massachusetts:Academic Press (2020). p. 107–28. 10.1016/bs.irn.2019.10.020 32204828

[14] Horvath S Raj K. DNA Methylation-based biomarkers and the epigenetic clock theory of ageing. Nat Rev Genet (2018) 19(6):371–84. doi: 10.1038/s41576-018-0004-3 29643443

[15] Dor Y Cedar H. Principles of DNA methylation and their implications for biology and medicine. Lancet. (2018) 392(10149):777–86. doi: 10.1016/S0140-6736(18)31268-6 30100054

[16] Chaix R Fagny M Cosin-Tomás M Alvarez-López M Lemee L Regnault B. Differential DNA methylation in experienced meditators after an intensive day of mindfulness-based practice: Implications for immune-related pathways. Brain Behavior Immunity. (2020) 84:36–44. doi: 10.1016/j.bbi.2019.11.003 31733290 PMC7010561

[17] Sae-Lee C Corsi S Barrow TM Kuhnle GGC Bollati V Mathers JC. Dietary intervention modifies DNA methylation age assessed by the epigenetic clock. Mol Nutr Food Res (2018) 62(23):e1800092. doi: 10.1002/mnfr.201800092 30350398

[18] Rentscher KE Klopack ET Crimmins EM Seeman TE Cole SW Carroll JE. Lower social support is associated with accelerated epigenetic aging: Results from the health and retirement study. medRxiv (2022). doi: 10.1101/2022.06.03.22275977

[19] Raffington L Belsky DW. Integrating DNA methylation measures of biological aging into social determinants of health research. Curr Environ Health Rep (2022) 9(2):196–210. doi: 10.1007/s40572-022-00338-8 35181865

[20] Schmitz LL Zhao W Ratliff SM Goodwin J Miao J Lu Q. The socioeconomic gradient in epigenetic ageing clocks: Evidence from the multi-ethnic study of atherosclerosis and the health and retirement study. Epigenetics. (2022) 17(6):589–611. doi: 10.1080/15592294.2021.1939479 34227900 PMC9235889

[21] Chen BH Marioni RE Colicino E Peters MJ Ward-Caviness CK Tsai PC. DNA Methylation-based measures of biological age: meta-analysis predicting time to death. Aging (Albany NY). (2016) 8(9):1844–65. doi: 10.18632/aging.101020 PMC5076441 27690265

[22] Yousefi PD Suderman M Langdon R Whitehurst O Davey Smith G Relton CL. DNA Methylation-based predictors of health: applications and statistical considerations. Nat Rev Genet (2022) 23:369–83. doi: 10.1038/s41576-022-00465-w 35304597

[23] Crimmins EM Thyagarajan B Levine ME Weir DR Faul J. Associations of age, sex, Race/Ethnicity, and education with 13 epigenetic clocks in a nationally representative U.S. sample: The health and retirement study. J Gerontology: Ser A (2021) 76(6):1117–23. doi: 10.1093/gerona/glab016 PMC8140049 33453106

[24] Ren JT Wang MX Su Y Tang LY Ren ZF. Decelerated DNA methylation age predicts poor prognosis of breast cancer. BMC cancer. (2018) 18(1):1–8. doi: 10.1186/s12885-018-4884-6 30333003 PMC6191915

[25] Kresovich JK Xu Z O’Brien KM Weinberg CR Sandler DP Taylor JA. Methylation-based biological age and breast cancer risk. JNCI: J Natl Cancer Institute. (2019) 111(10):1051–8. doi: 10.1093/jnci/djz020 PMC6792078 30794318

[26] Rozenblit M Hofstatter E Liu Z O’Meara T Storniolo AM Dalela D. Evidence of accelerated epigenetic aging of breast tissues in patients with breast cancer is driven by CpGs associated with polycomb-related genes. Clin epigenetics. (2022) 14(1):1–1. doi: 10.1186/s13148-022-01249-z 35209953 PMC8876160

[27] Kresovich JK Xu Z O’Brien KM Weinberg CR Sandler DP Taylor JA. Epigenetic mortality predictors and incidence of breast cancer. Aging (Albany NY). (2019) 11(24):11975–87. doi: 10.18632/aging.102523 PMC6949084 31848323

[28] Hofstatter EW Horvath S Dalela D Gupta P Chagpar AB Wali VB. Increased epigenetic age in normal breast tissue from luminal breast cancer patients. Clin Epigenet (2018) 10(1):112. doi: 10.1186/s13148-018-0534-8 PMC6114717 30157950

[29] Coughlin SS. Social determinants of breast cancer risk, stage, and survival. Breast Cancer Res Treat (2019) 177(3):537–48. doi: 10.1007/s10549-019-05340-7 31270761

[30] Valencia CI Gachupin FC Molina Y Batai K. Interrogating patterns of cancer disparities by expanding the social determinants of health framework to include biological pathways of social experiences. Int J Environ Res Public Health (2022) 19(4):2455. doi: 10.3390/ijerph19042455 35206642 PMC8872134

[31] Jung SE Shin KJ Lee HY. DNA Methylation-based age prediction from various tissues and body fluids. BMB Rep (2017) 50(11):546. doi: 10.5483/BMBRep.2017.50.11.175 28946940 PMC5720467

[32] El Khoudary SR Greendale G Crawford SL Avis NE Brooks MM Thurston RC. The menopause transition and women's health at midlife: a progress report from the study of women's health across the nation (SWAN). Menopause (New York NY). (2019) 26(10):1213. doi: 10.1097/GME.0000000000001424 PMC6784846 31568098

[33] Levine ME Lu AT Chen BH Hernandez DG Singleton AB Ferrucci L. Menopause accelerates biological aging. Proc Natl Acad Sci U S A. (2016) 113(33):9327–32. doi: 10.1073/pnas.1604558113 PMC4995944 27457926

[34] Harlow SD Burnett-Bowie SA Greendale GA Avis NE Reeves AN Richards TR. Disparities in reproductive aging and midlife health between black and white women: The study of women’s health across the nation (SWAN). Women's Midlife Health (2022) 8(1):1–7. doi: 10.1186/s40695-022-00073-y 35130984 PMC8822825

[35] El Khoudary SR Aggarwal B Beckie TM Hodis HN Johnson AE Langer RD. Menopause transition and cardiovascular disease risk: implications for timing of early prevention: a scientific statement from the American heart association. Circulation. (2020) 142(25):e506–32. doi: 10.1161/CIR.0000000000000912 33251828

[36] Lundgren S Kuitunen S Pietiläinen KH Hurme M Kähönen M Männistö S. BMI is positively associated with accelerated epigenetic aging in twin pairs discordant for body mass index. J Intern Med (2022) 292:627–640. doi: 10.1111/joim.13528 35699258 PMC9540898

[37] Jatoi I Sung H Jemal A. The emergence of the racial disparity in US breast-cancer mortality. New Engl J Med (2022) 386(25):2349–52. doi: 10.1056/NEJMp2200244 35713541

[38] Fejerman L Ramirez AG Nápoles AM Gomez SL Stern MC. Cancer epidemiology in Hispanic populations: What have we learned and where do we need to make progress? Cancer Epidemiol Biomarkers Prev (2022) 31(5):932–41. doi: 10.1158/1055-9965.EPI-21-1303 PMC9081152 35247883

[39] Yedjou CG Sims JN Miele L Noubissi F Lowe L Fonseca DD. Health and racial disparity in breast cancer. Adv Exp Med Biol (2019) 1152:31–49. doi: 10.1007/978-3-030-20301-6_3 31456178 PMC6941147

[40] López-Otín C Pietrocola F Roiz-Valle D Galluzzi L Kroemer G. Meta-hallmarks of aging and cancer. Cell Metab (2023) 35(1):12–35. doi: 10.1016/j.cmet.2022.11.001 36599298

[41] Pellicano GR Daniela S Chiara C Arianna G Paola A Carlo L. Epigenetic correlates of the psychological interventions outcomes: A systematic review and meta-analysis. J Affect Disord Rep (2022) 7:100310. doi: 10.1016/j.jadr.2022.100310

[42] Nevalainen T Kananen L Marttila S Jylhävä J Mononen N Kähönen M. Obesity accelerates epigenetic aging in middle-aged but not in elderly individuals. Clin Epigenet (2017) 9:20. doi: 10.1186/s13148-016-0301-7 PMC5310016 28289477

